# Brassinolide Increases Potato Root Growth* In Vitro* in a Dose-Dependent Way and Alleviates Salinity Stress

**DOI:** 10.1155/2016/8231873

**Published:** 2016-10-10

**Authors:** Yueqing Hu, Shitou Xia, Yi Su, Huiqun Wang, Weigui Luo, Shengying Su, Langtao Xiao

**Affiliations:** ^1^Hunan Provincial Key Laboratory of Phytohormones and Growth Development, College of Bioscience and Biotechnology, Hunan Agricultural University, Changsha 410128, China; ^2^School of Life Science and Environmental Resource, Yichun University, Yichun 336000, China

## Abstract

Brassinosteroids (BRs) are steroidal phytohormones that regulate various physiological processes, such as root development and stress tolerance. In the present study, we showed that brassinolide (BL) affects potato root* in vitro* growth in a dose-dependent manner. Low BL concentrations (0.1 and 0.01 *μ*g/L) promoted root elongation and lateral root development, whereas high BL concentrations (1–100 *μ*g/L) inhibited root elongation. There was a significant (*P* < 0.05) positive correlation between root activity and BL concentrations within a range from 0.01 to 100 *μ*g/L, with the peak activity of 8.238 mg TTC·g^−1^ FW·h^−1^ at a BL concentration of 100 *μ*g/L. Furthermore, plants treated with 50 *μ*g/L BL showed enhanced salt stress tolerance through* in vitro* growth. Under this scenario, BL treatment enhanced the proline content and antioxidant enzymes' (superoxide dismutase, peroxidase, and catalase) activity and reduced malondialdehyde content in potato shoots. Application of BL maintain K^+^ and Na^+^ homeostasis by improving tissue K^+^/Na^+^ ratio. Therefore, we suggested that the effects of BL on root development from stem fragments explants as well as on primary root development are dose-dependent and that BL application alleviates salt stress on potato by improving root activity, root/shoot ratio, and antioxidative capacity in shoots and maintaining K^+^/Na^+^ homeostasis in potato shoots and roots.

## 1. Introduction

Roots are fundamentally important for plant growth and survival because of their essential roles in water and nutrient uptake. As a serious stressful factor, salinity leads to growth arrest and crop yields decline [1]. Besides reduced nutrient uptake and adverse effects of growth and development, salinity stress can also result in osmotic stress and ionic imbalance [2]. Exposure to salinity enhances oxidative stress and the overproduction of reactive oxygen species (ROS), resulting in plant damage. ROS such as superoxide radical (O_2_^•−^), hydroxyl radical (OH^•^), hydrogen peroxide (H_2_O_2_), and singlet oxygen (^1^O_2_) are highly reactive under NaCl stress and can alter normal cell metabolism through oxidative damage of membranes by lipid peroxidation [3]. To protect against oxidative damage, plants have evolved a complex antioxidant defense system which includes nonenzymatic antioxidants, such as glutathione, carotenoids, and flavonoids and several enzymatic antioxidants, including superoxide dismutase (SOD), catalase (CAT), and peroxidases (POD) [4]. SOD is an intracellular antioxidant enzyme which combats oxidative stress by catalyzing the conversion of superoxide to H_2_O_2_ in organelles and cytosol. CAT scavenges H_2_O_2_ generated in photorespiration, which dismutates H_2_O_2_ into water and O_2_. POD possess broad specificities located in vacuoles, cell walls, and the cytosol, which consumes H_2_O_2_ by catalyzing H_2_O_2_ to decompose other substrates [2, 5, 6]. Proline (Pro) is a major osmoregulatory substance and also known as a substance with nonenzymatic antioxidant properties [7]. It was reported that Pro might contribute to plant adaptive responses to salinity by regulating K^+^ transport across the plasma membrane in barley [8] and exogenously supplied proline significantly reduced NaCl-induced K^+^ efflux from barley roots in a dose-response manner and thus resulted in better K^+^ in roots [9]. Malondialdehyde (MDA) content indicates the damage caused by ROS [10]. Salinity leads to imbalanced ion ratio results from high Na^+^ and Cl^−^ concentrations that are detrimental to plants, or decline of tissue K^+^ content which is central to normal cell metabolism, or both [11, 12]. Numerous studies have reported that regulation of K^+^ homoeostasis is a common denominator of plant adaptive responses to stress environment including drought, salinity, and oxidative stress [12, 13]. Thus, not only maintenance of a high K^+^/Na^+^ ratio in cytosol but also retention of absolute concentrations of K^+^ is a biochemical strategy for plants growth in a saline environment.

Brassinosteroids (BRs), a group of naturally occurring steroidal compounds in plants, contribute to growth, vascular differentiation, development [14], and response to biotic and abiotic stresses [15–19]. These effects are mediated by BR-induced genes, including defense genes and genes that can detoxify ROS produced in plants experiencing abiotic stress [15]. Exogenous BR application enhanced salinity stress tolerance enhancement in a variety of crop species [14, 20]. Treatment with 24-epibrassinolide (EBL) reduced the salt-stress-induced inhibition of seed germination in* Arabidopsis thaliana*,* Brassica napus*, and* Cucumis sativus* [21, 22]. Presowing seeds with BR increased the growth vigor of* Medicago sativa* seedlings under saline stress [23] and reduced the deleterious effects caused by saline stress in* Pisum sativum* and* Cicer arietinum* [24, 25]. The application of EBL to leaves enhanced the photosynthetic capacity and regulated antioxidant enzymes of salt-stressed wheat, thereby increasing plant biomass and leaf area per plant [26]. Also, treatment with EBL downregulated the gene expression of* OsDWF4* and* SalT*, upregulated the gene expression of* OsBRI1*, and enhanced Pro content and antioxidant enzymes' activity under salinity stress in rice [27].

As an important staple crop in China, potato displays high sensitivity to excess soil salinity. Since* in vitro* culture systems have been successfully applied to produce virus-free seed potatoes, they could also be used to screen for salt tolerance. Although many studies demonstrated a positive effect of BRs application on plant tolerance to salt stresses [20, 28, 29], BRs stimulate root growth at low concentrations but are inhibitory at higher concentrations [30–32]. The roles of BRs, however, in adventitious root growth are rarely understood in tissue culture systems. Thus, it is necessary to determine the effects and optimal concentration of BRs for potato root formation and plant growth throughout the process of* in vitro* culture. In our study, an* in vitro* tissue culture system was used to examine the effects of brassinolide (BL) on the resistance of potatoes to salt stress. We focused on the physiological, K^+^/Na^+^ homeostasis and characterization of the antioxidant capacity of BL-treated plantlets cultivated* in vitro* under salt stress. The growth parameters, root activity, accumulation of MDA and Pro, the typical antioxidant enzymes' (SOD, CAT, and POD) activities, and mineral tissue (Na^+^, K^+^) content were evaluated for plantlets' tolerance to salt stress.

## 2. Materials and Methods

### 2.1. Plant Material and Experimental Treatments

Potatoes (*Solanum tuberosum* L. “Hui 2”) were propagated* in vitro* from single-node cuttings on standard Murashige and Skoog (MS) medium containing 25 g/L sucrose solidified with 0.7% (w/v) agar. They were grown in a growth chamber under a 16 h photoperiod at 22°C. These plants were harvested 4 weeks later and used for subsequence experiments.

For root growth assays, MS medium was supplemented with 0, 0.01, 0.1, 1, 10, and 100 *μ*g/L BL (Sigma Chemicals, USA). After 20 days, the root length (measured using vernier caliper), numbers of adventitious roots, root activity, and plant biomass were measured.

To examine the physiological role of BL in salinity stress, a 2-factor salinity and BL randomized block design was employed. Four levels (0, 50, 75, and 100 mM) for salinity (NaCl, Sigma Chemicals, USA) and 2 levels for BL (0, 50 *μ*g/L) were designed. After 31 days of growth, 24 plants per treatment were collected to determine the plant biomass for shoots and roots. The stem fresh weight, stem length, root fresh weight, root length, and root activity were also measured. The remaining fresh samples were used for physiological analysis and determination of tissue K^+^ and Na^+^ content.

### 2.2. Root Vigor Assessments

Root activity in terms of 2,3,5-triphenyl tetrazolium chloride (TTC) reduction was measured as described previously [33]. About 0.5 g of fresh root samples were incubated for 2 h in a mixture of 5 mL 0.4% (w/v) TTC and 5 mL phosphate buffer (pH 7.5) at 37°C. The assays were terminated by adding 2 mL 1 M sulfuric acid to the reaction mixture. For triphenylformazan (TTCH, red product) extraction, the roots were removed and blotted dry on filter paper and ground in a mortar containing 3-4 mL ethyl acetate. The liquid phase was then transferred to a 10 mL stoppered test tube. Ethyl acetate was added to the 10 mL level, and the released TTCH was quantified photometrically at 485 nm. The TTCH reduction was calculated based on the slopes of the obtained curves, which were corrected for assay background slopes from “no-extract controls.” The OD values were used to calculate the equivalent TTCH concentrations to determine the root vigor for each fresh root weight as follows: root vigor (TTCH *μ*g·g^−1^ FW·h^−1^) = TTCH reduction (TTCH *μ*g)/fresh weight (FW g)/time (h).

### 2.3. Measurement of MDA and Pro Content

Lipid peroxidation was estimated by measuring the level of MDA production using the thiobarbituric acid (TBA) method as described by Hodges et al. [34]. Fresh shoot samples (0.15 g) were crushed by grinding in a mortar containing 4.5 mL 10% TCA, after which the homogenized material was centrifuged at 5,000 ×g for 15 min at 4°C. The supernatant was transferred to a centrifuge tube and volume (V) was recorded. Then, 2 mL of supernatant was mixed with 2 mL 0.6% TBA, the homogenizing mixture was heated in boiling water for 20 min, and the reaction was terminated in an ice bath followed by the centrifugation at 5,000 g for 10 min. Approximately 2 mL (V1) of the supernatant was transferred to a cuvette. The absorbance of the supernatant was determined at 450, 532, and 600 nm, respectively. The MDA content was determined as follows:

(1) MDA  concentration μmol/L=6.45∗A532−A600−0.56∗A450,MDA  content μmol/g FW=Cμmol/L∗VL∗V1mL/2 mL∗Mg FW.

The Pro content was estimated following the procedure given by Bates et al. [35]. Fresh shoot samples (0.15 g) were homogenized with 4.5 mL 3% (w/v) sulfosalicylic acid, and the homogenate was heated in a boiling water bath for 30 min then filtered through 0.2 *μ*m filter paper, and the extract volume was marked as Vt. The supernatant was used for the Pro estimation. The reaction mixture was composed of 2 mL of plant extract and an equal volume of glacial acetic acid and acid ninhydrin. The test tubes containing the above mixture were heated in a boiling water bath for 30 min. The reaction was terminated in an ice bath followed by the addition of 4 mL of toluene. The contents were shaken vigorously and allowed to separate into phases. The chromophase containing the upper toluene phase with volume marked as V was carefully separated using a pipette; the absorbance was noted at 520 nm. The amount of Pro was calculated from the standard curve and expressed as *μ*g·g^−1^ FW. The proline content was determined as follows: proline content (*μ*g/gFW) = C*∗*Vt/(V*∗*W).

### 2.4. Determination of SOD, CAT, and POD

To estimate antioxidant activities, frozen shoots (about 0.5 g) were homogenized (1 : 9 w/v) in 0.1 M cooled phosphate buffer (pH 7.8) or normal saline. The homogeneous mixture was centrifuged at 12000 ×g for 20 min at 4°C and the supernatants were collected for determining antioxidase activity.

The activities of POD, CAT, and SOD in the shoot homogenate were examined by a reagent kit (Nanjing Jiancheng Bioengineering Institute, Nanjing, China). The principles of these kits are briefly described as follows.

POD activity was measured according to the reaction of hydrogen peroxide catalysis by POD, and we detected the changes of absorbance at 420 nm and calculated the POD activity. One unit of POD activity was defined as the amount of the enzyme in 1 g of fresh tissue that reduced 1 *μ*g of H_2_O_2_ per minute at 37°C. CAT activity was measured according to the ammonium molybdate spectrophotometric method, where ammonium molybdate rapidly terminated the H_2_O_2_ degradation reaction catalyzed by CAT and reacted with the residual H_2_O_2_ to generate a yellow complex, which could be monitored by absorbance at 405 nm. One unit of catalase activity was defined as the amount of enzyme in 1 g of fresh tissue that reduced 1 *μ*mol of H_2_O_2_ per minute at 37°C. SOD activity was determined using the xanthine oxidase method, based on its ability to inhibit the oxidation of hydroxylamine by the xanthine-xanthine oxidase system. One unit of SOD activity was defined as the amount of the enzyme inhibiting the oxidation by 50%.

### 2.5. Measurement of Na^+^ and K^+^ Concentrations

The root and shoot tissues were harvested from the two-week-old potato plants grown under salt stress and the control, washed with distilled water for 2 times, dried at 65°C for 48 hours, and digested in HNO_3_-HClO_4_ solution. The final extracts volume was adjusted to 50 mL with 2% HNO_3_ and filtered before the assay. Na^+^ and K^+^ concentrations were determined by using inductively coupled plasma-mass spectrometry (ICP-MS, Agilent Technologies 7700x, Waldbronn, Germany) as previously described [36].

### 2.6. Data Analysis

All statistical analyses were performed with SPSS 20.0. Treatment means were separated using Student's *t* test or Duncan's new multiple range test at 95% or 99% level of probability. Microsoft Excel 2010 and Microsoft Office Visio 2010 (Microsoft Corporation, USA) were used to generate graphs.

## 3. Results

### 3.1. The Effects of BR on Root Development Are Dose-Dependent

**Figure 1:**
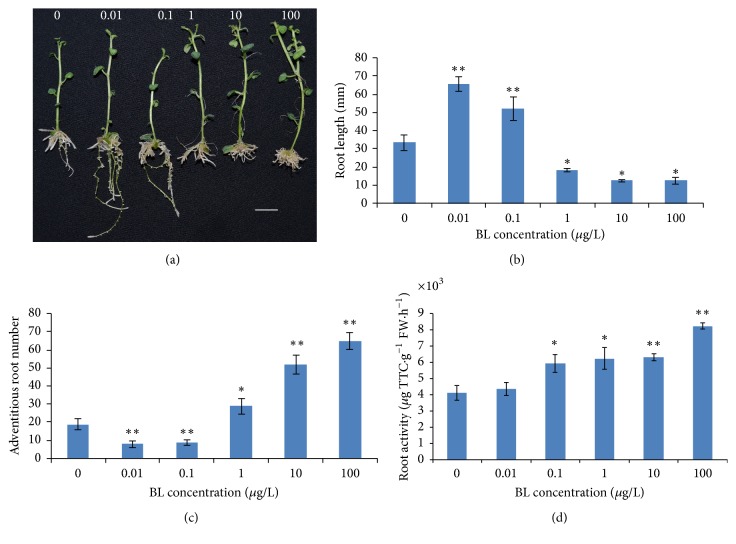
Brassinolide (BL) effect on the adventitious root growth of potatoes. (a) Phenotype of 20-day-old plants from left to right control (0 *μ*g/L) and treated with 0.01, 0.1, 1, 10, and 100 *μ*g/L BL. (b) Root-length measurements of various concentrations (*μ*g/L) of BL-treated plants compared with that of the control. Values represent the mean of 43 measurements ± SD. (c) Adventitious roots numbers in BL-treated plantlets at different concentrations (*μ*g/L) compared with that of control. Values represent the mean of 24 measurements ± SD. (d) Root activity of BL-treated plants at different concentrations (*μ*g/L) compared with that of the control. Values represent mean ± SD of three biological replicates; asterisks indicate significant differences from the control: *∗* and *∗∗* indicate significance at 0.05 and 0.01, respectively.

BL promoted root growth significantly only at low concentrations (0.01 and 0.1 *μ*g/L) in 20-day-old plantlets, and BL concentrations higher than 1 *μ*g/L inhibited root elongation (Figure 1(a)). The length of roots of 20-day-old plantlets treated with 100 *μ*g/L BL was lower ~53% than untreated roots of the* in vitro* cuttings and increased ~96% when treated with 0.01 *μ*g/L BL (Figure 1(b)). BL-induced root shortening was coupled to a significant increase in adventitious roots number (Figure 1(c)) and root activity (Figure 1(d); *P* < 0.05 or 0.01). Although lower BL concentration promoted adventitious roots length growth, the number of adventitious roots decreased. However, when BL at higher concentrations inhibited the growth of adventitious root length, more adventitious roots were developed. Under the treatment of 0.01, 0.1, 1, 10, and 100 *μ*g/L BL, the number of adventitious roots was 58 and 53% lower and 53, 174, and 242% higher than the control, respectively.

Generally, the root activity of all BL-treated plantlets increased with BL concentration. A maximum increase in root activity was observed in plants treated with 100 *μ*g/L BL. Compared to those treated without BL (control), root activity at 0.01, 0.1, 1, 10, and 100 *μ*g/L BL treatment increased by 6, 44, 52, 54, and 100%, respectively. The results show that BL can affect* in vitro* potato root growth in a dose-dependent manner and the application of BL can increase root activity and adventitious roots number.

Moreover, total plant fresh weight of all treatments except 0.01 *μ*g/L BL showed a significant increase (Table 1; *P* < 0.05) compared to that of the control. As BL improved root absorbing ability and biomass of* in vitro* potatoes, it was of interest to determine the salinity response when BL was applied to MS medium. Given that significant increase of root weight, shoot weight, and root/shoot ratio was observed only at high concentrations of 100 and 10 *μ*g/L BL (Table 1; *P* < 0.05), we applied 50 *μ*g/L BL to carry out salt stress experiments.

### 3.2. BL Increases the Biomass of NaCl-Stressed Potato Plants

**Figure 2:**
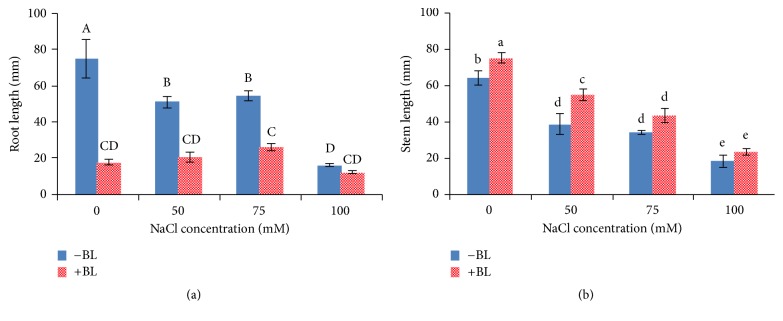
Effects of 50 *μ*g/L BL on the growth of NaCl-stressed potato plants. (a) Root length and (b) stem length under 50, 75, and 100 mM NaCl and the control. Columns marked with different capital letters indicate significant differences by Duncan's new multiple range test at *P* < 0.01. Columns marked with different lowercase letters indicate significant difference at *P* < 0.05.

NaCl stress severely inhibited* in vitro* potato plantlets growth (Table 2 and Figure 2). The inhibition was more pronounced in roots than shoots, causing an overall decline in the root/shoot fresh weight ratio, whereas it showed the second-highest value in the treatment of 75 mM NaCl for the development of more and thicker adventitious roots. Compared with the control, application of NaCl at different concentrations (50, 75, and 100 mM) showed a distinct adverse effect on growth parameters of potato plantlets, such as shoot length, root length, and fresh biomass accumulation (Table 2 and Figure 2). Application of 50 *μ*g/L BL increased the biomass of roots and shoots and increased the root/shoot fresh weight ratio via inducing more adventitious roots. The maximum enhanced effect of BL-treated plantlets weight in roots, shoots, and total plants was 75.8 (0 mM NaCl), 35.9 (75 mM NaCl), and 39.3% (75 mM NaCl), respectively. NaCl decreased the length of stems and roots as compared to the control; however, it induced more and longer roots with the treatment of 75 mM NaCl than that of 50 mM NaCl. The 50 *μ*g/L BL treatment increased the plant height but decreased the root length.

### 3.3. BL Increases Pro Content and Decreases MDA Content

**Figure 3:**
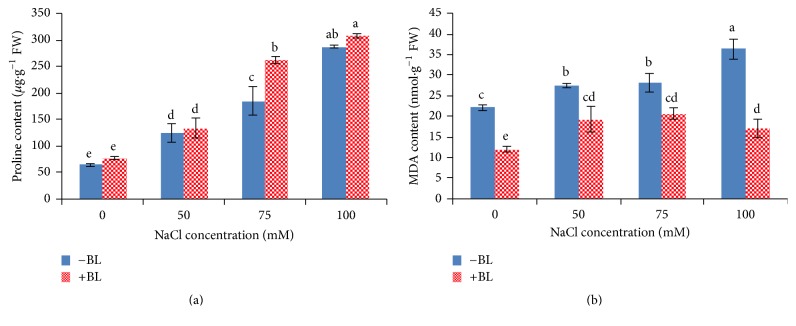
Effects of 50 *μ*g/L BL on Pro (a) and MDA (b) content of potato shoots under salt stress conditions of 0 (control), 50, 75, and 100 mM NaCl. Columns marked with different lowercase letters are significantly different as determined by Duncan's new multiple range test at *P* < 0.05.

Increasing the salt concentration significantly (*P* < 0.05) affected Pro and MDA content of shoots (Figure 3). A continuous increase in Pro content occurred with enhanced salinity level (Figure 3(a)) with a sharp increase of 343% at 100 mM NaCl. Application of 50 *μ*g/L BL to MS medium enhanced Pro content, but a significant (*P* < 0.05) increase of Pro content was observed at high concentrations of 75 mM NaCl, and the maximum 41.6% increase was also observed at 75 mM NaCl. A similar trend in the MDA content occurred with increasing concentrations of NaCl. The MDA content was elevated to more than 164% at 100 mM NaCl than that of control plantlets. BL application significantly reduced the amount of MDA in the control and salt-stressed plantlets; however, the value in the salt-stressed plantlets was higher than that of the control. A maximum of 53% decline in MDA content was observed in plantlets growing in the combination of 50 *μ*g/L BL + 100 mM NaCl than those growing in 100 mM NaCl alone.

### 3.4. BL Enhances Antioxidant Enzyme' Activity

**Figure 4:**
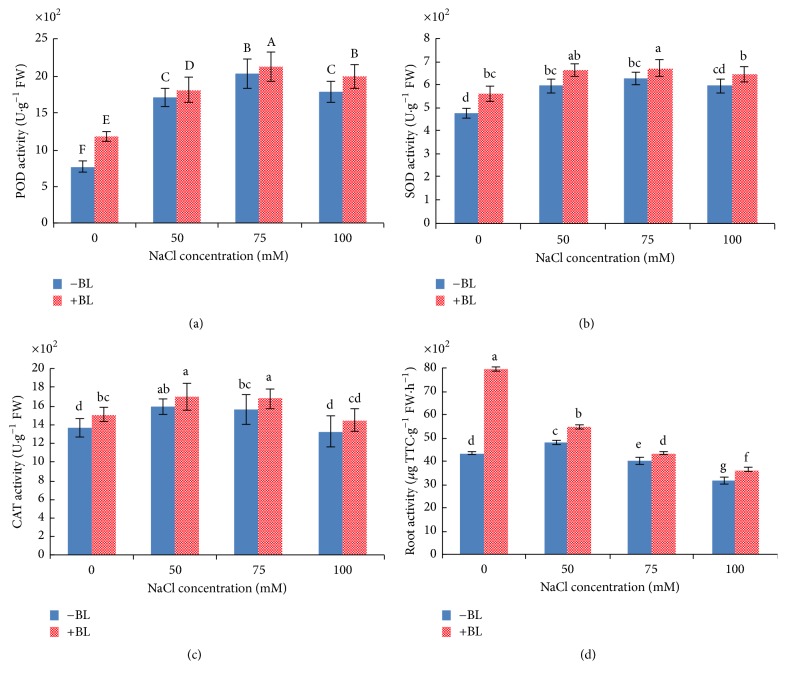
Effects of 50 *μ*g/L BL on POD (a), SOD (b), CAT (c), and root activity (d) of potato shoots under salt stress conditions of 0, 50, 75, and 100 mM NaCl. Columns marked with different lowercase letters are significantly different by Duncan's new multiple range test at *P* < 0.05. Columns marked with different capital letters are significantly different by Duncan's new multiple range test at *P* < 0.01.

In the present study, salt stress generally enhanced the activity of the typical antioxidant enzymes (Figure 4), whereas different antioxidant enzymes showed slight variations. Our results showed a significant increase (*P* < 0.05) in SOD activity in plants growing in 50, 75, and 100 mM NaCl compared to that of the control (Figure 4(b)). SOD, similar to POD and CAT activity, decreased at 100 mM NaCl but was still higher than that of the control. Treatment with BL resulted in significant increase in SOD activity at all concentrations but 50 mM NaCl.

An increase in POD (Figure 4(a)) and CAT (Figure 4(c)) activity is a typical response observed in plants grown under increasing of NaCl concentration, whereas, at 100 mM NaCl, activities of both enzymes declined and CAT activity decreased more sharply. CAT activity of 75 mM NaCl-stressed plants decreased less than that of 50 mM NaCl-stressed plants. Application of BL resulted in a considerable enhancement in the activity of both antioxidant enzymes.

### 3.5. BL Enhances Root Activity of NaCl-Stressed Potato Plants

NaCl stress significantly (*P* < 0.05) decreased root activity at 75 and 100 mM NaCl but significantly (*P* < 0.05) increased it at 50 mM NaCl (Figure 4(d)). The application of BL to MS medium enhanced the root activity in the control and in NaCl-stressed plants; and a maximum increase of 83% was observed under plants treated with BL only as compared to control plants. Compared with NaCl treatment alone, root activity of BL + NaCl treatment increased by 13, 6, and 14%, respectively. The results show that the influence of BL on root activity of NaCl-stressed plants is lower than that on the control.

### 3.6. BL Affects Na^+^ and K^+^ Homeostasis of Potato Plants under Salinity

**Figure 5:**
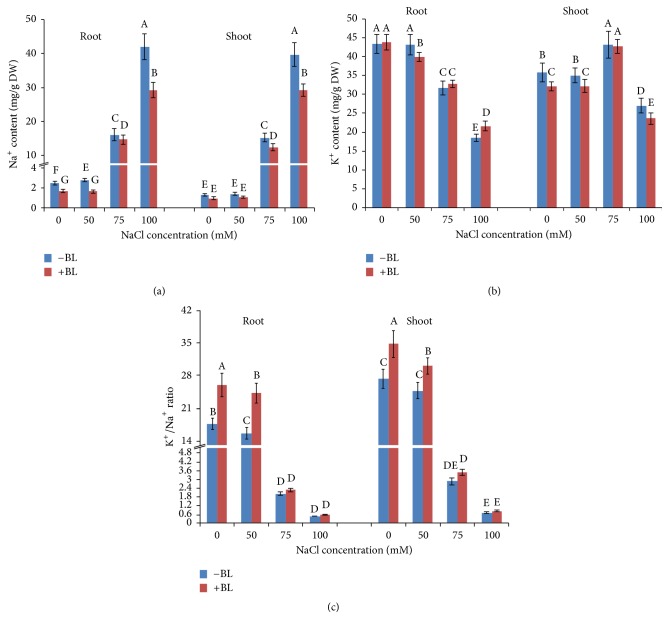
Effects of 50 *μ*g/L BL on Na^+^ (a) and K^+^ (b) content and K^+^/Na^+^ ratio (c) of* in vitro* potato root and shoot under different NaCl treatments and the control for 20 days. Columns marked with different letters are significantly different by Duncan's new multiple range test at *P* < 0.05.

To investigate the effect of BL on biochemical mechanism of ion homeostasis to salinity tolerance, we measured the concentrations of Na^+^ and K^+^ accumulation in tissues and also calculated the K^+^/Na^+^ ratio in potato roots and shoots, respectively. With the increase of the NaCl concentration, Na^+^ accumulation exhibited a significant increase in both roots and shoots of potato plants especially in high concentration of NaCl. Under 75 and 100 mM NaCl, the roots Na^+^ concentrations were 6.5-fold and 17.1-fold higher than that of control in roots and were 11.0-fold and 30.25-fold higher than that of control in shoots, respectively (Figure 5(a)). K^+^ accumulations in both roots and shoots of* in vitro* potato were reduced under salt stress (except K^+^ content in shoots under 75 mM NaCl); however, the decline of K^+^ content was more moderate compared to the increase of Na^+^ accumulation, and especially in shoots, K^+^ accumulations were even higher than that of control level under 75 mM NaCl. Correspondingly, more Na^+^ accumulation resulted in a significant decrease of K^+^/Na^+^ ratio in both roots and shoots under various NaCl treatments. Application of 50 *μ*g/L BL significantly decreased roots Na^+^ content of salt-treated and nontreated plants under saline conditions, while the decline in shoots under control and 50 mM NaCl was not significant. The effect of BL on K^+^ accumulations showed more variations; significant increase and decrease were observed in tissue (Figure 5(b)). Compared with NaCl treatment alone, BL + NaCl treatment increased K^+^/Na^+^ ratio under 0, 50, 75, and 100 mM NaCl to 47.6, 55.9, 12.6, and 22.7% in roots and to 27.7, 21.4, 21.1, and 19.1% in shoots, respectively (Figure 5(c)). These results indicated that exogenous BL application reduced Na^+^ accumulation and improved K^+^/Na^+^ ratio in salt-stressed tissue.

## 4. Discussion

### 4.1. BL Induced a Well-Developed Root System and Improved Root Absorption

In the present study, we demonstrated that the effects on* in vitro* potato adventitious root formation are strongly dependent on the BL concentration. Exogenous BL stimulated root growth at low concentrations (0.01 and 0.1 *μ*g/L). Inhibition of root growth occurred at higher BR levels, which exceed a certain level of 1 *μ*g/L. The BL dose-response phenotype in adventitious root development of potatoes was consistent with previous studies of seed root growth of* A. thaliana* [31]. The genetic analysis showed that both loss-of-function and gain-of-function BR-related mutants in* A. thaliana* have reduced meristem size and the dose-response analysis revealed that BRs promoted the exit of cells from the meristematic region and indicated that BRs promote cell elongation at the meristematic zone of the root. Thus, balanced BR signaling is required to maintain normal root growth rates through the control of the root meristem size. The root became curved at 1 to 100 *μ*g/L BL, which could have resulted from unbalanced BL signaling inhibiting cell division and gravitropism.

Another typical enhanced growth phenotype of shoot (data not shown) was accompanied by inhibition of root growth; thus, measurement of the root activity, adventitious roots numbers, and plant biomass were carried out. The results showed that increasing BL concentrations enhanced root activity and adventitious roots number. Under 0.01 and 0.1 *μ*g/L BL-treatments, there were fewer adventitious roots than that of the control, whereas a well-developed root system was formed, with more lateral roots (data not shown) developing at the adventitious roots. These results concur with previous studies that showed in lateral root growth of* A. thaliana* [37]. In their study, exogenous BR promoted DR5::GUS expression in the root tips and lateral root development by increasing acropetal auxin transport. Thus, BRs are required for lateral or adventitious root development and BRs act synergistically with auxin to promote lateral/adventitious root formation. As one of the important physiological indexes of plant growth, root activity affects the growth and yield of overground portion [33]. Accordingly, increasing root activity with BL might result in enhanced root physiology.

### 4.2. BL Alleviated Salinity Injury of Potato* In Vitro*

The development of salt-tolerant crops is fundamental to alleviate salinity disadvantageous effect. However, achievements have been limited, due to complexity of the salt tolerance trait. Thus, understanding the physiological processes involved in salt tolerance is essential to improve the salt tolerance and develop biochemical strategies to enhance crop yields under salinity stress. The first* in vitro* selection of salt stress tolerant plant was reported in* Nicotiana sylvestris* [38]. Several studies have shown that BRs enhance plant tolerance and boost crop yield to a variety of environmental stresses and species [14, 22, 28, 39, 40]. Our results demonstrated that BL enhanced the biomass of NaCl-stressed potato plants and BL application enhanced the root/shoot ratio. The enhancement in biomass resulted from longer stem length and a well-developed root system. The longer stem length phenotype was consistent with the contribution of BRs in promoting the cell expansion of shoot organs [41]. NaCl resulted in a sharp overall reduction of growth indicators such as shoot height, root length, root fresh weight, shoot fresh weight, and root/shoot ratio; however, the value of root fresh weight and root/shoot ratio was higher at 75 than 50 mM NaCl. This is because longer and more adventitious roots were developed at 75 mM NaCl than at 50 mM NaCl. Application of BL alleviated salinity injury of potato* in vitro* by enhancing the root/shoot ratio, shoot length, shoot weight, root weight, and biomass.

To reveal physiological processes, we measured the content of Pro, MDA, root activity, and antioxidant enzymes' activity. Exogenous application of BL only significantly enhanced Pro content of NaCl-stressed potato plants under 75 mM NaCl. Thus, the osmotic adjustment may not achieve much by enhancing the production of osmoregulatory compounds of potatoes under salinity stress. Given the fact that accumulation of K^+^ plays a pivotal role in this process, contributing on average between 35 and 50% of the cell osmotic potential in crops [13], we determined the tissue K^+^ content. A continuous increase in the level of MDA content was observed with increased salinity levels, and application of BL sharply decreased the MDA content. Enhanced MDA content indicates damage caused by ROS. It is necessary for ROS induced by NaCl to be scavenged by antioxidant enzymes for plant survival. The resulting decrease in MDA levels after BL treatment could therefore indicate the efficiency of BL-induced scavenging of ROS as a result of enhanced antioxidant enzymes' activity. These data suggest the adaptation and salt stress tolerance of plants with BL application. Various antioxidant enzymes help in maintaining the balance in ROS production and scavenging and it is believed that enhanced activity of these enzymes facilitates stress protection. A continuous increase in antioxidant enzymes' activity occurred with enhanced salinity level; however, antioxidant enzymes' activity declined at 100 mM NaCl; therefore, it is possible that the high NaCl concentration had severely damaged the lipid membrane, which was confirmed by the high MDA content. BL application resulted in an overall enhancement in the activities of SOD, POD, and CAT under varying degrees of salt stress, suggesting the presence of an effective scavenging mechanism to remove ROS from the plant system and a potential mechanism of plant salt tolerance. Similar results were reported for antioxidant enzymes' activity [20, 29, 42], while some researches showed that there was no or even negative correlation between antioxidant enzymes' activity and salinity stress tolerance [4, 43]. The variation may be caused by the specific variety, tissue, sampling time/observation period, and culture conditions. To make the conclusion more convincing, the amounts of Na^+^ and K^+^ accumulated in roots and shoots were measured.

Maintaining constant intracellular ion homeostasis, especially K^+^ and Na^+^ homeostasis, is crucial for plant adapting to saline environments [44]. In this study, the potato showed significant increase in accumulation of Na^+^ and overall decline of K^+^ in both roots and shoots under NaCl stress. Application of BL improved Na^+^ exclusion in both shoots and roots of NaCl treated and nontreated plants, while the amounts of K^+^ did not increase in all situations. Similar results were also observed in* Gossypium hirsutum* [20]. K^+^/Na^+^ ratio also increased when exogenous BL was applied, while it remains a very low value (0.44–0.81) under 100 mM NaCl compared to control plants. In fact, the growth of explant was seriously inhibited under 100 mM NaCl, and it takes a long time of more than 15 days to develop adventitious root, while the average time for NaCl nontreated plants is about 5 days, and two-thirds of the plants develop etiolation symptom. The serious inhibition of high NaCl caused by significant decline in K^+^ content and K^+^/Na^+^ ratio concurred with previous studies that ion exclusion mechanism can provide a degree of tolerance to relatively low concentrations of NaCl but will not work at high concentrations of salt [45].

Roots uptake water and all inorganic nutrients for the plant and are the first organs to be affected by salinity stress; therefore, adaptation of roots to salt stress affects shoot response, physiological functions, and plant growth. BL-induced stress tolerance is a relatively complex process and involves the dynamics of several intrinsic factors. Thus, the growth-promoting effect of BL on salt-stressed potatoes could be attributed to its role in regulating osmotic pressure, ion homeostasis, lipid peroxidation levels, antioxidant enzymes activity, root activity, and adventitious root development. These results are important for the development of salinity-tolerant strains of potatoes that could be produced in more diverse environments, thereby enhancing global potato production.

## 5. Conclusion

The results presented in this report have clearly shown dose-dependent effect of BL on* in vitro* potato adventitious root growth. Although application of 1–100 *μ*g/L BL inhibited the growth of root length, root number and biomass increased and root activity and root/shoot ratio improved. Thus, we applied high concentration of 50 *μ*g/L BL to* in vitro* potato under salt stress. BL application resulted in an increased physiological action by maintaining K^+^/Na^+^ homeostasis in shoots and roots, improving root activity, root/shoot ratio, and antioxidative capacity in shoots.

## Figures and Tables

**Table 1:** Effect of BL on biomass accumulation and root/shoot ratio of *in vitro* potato plants.

Treatments	Biomass accumulation (g)			Relative biomass (%)	Root/shoot ratio
	Root fresh weight	Shoot fresh weight	Total plant fresh weight		
0 *μ*g/L BL	0.056 ± 0.004	0.129 ± 0.016	0.185 ± 0.011	100	0.437 ± 0.080
0.01 *μ*g/L BL	0.064 ± 0.012	0.143 ± 0.029	0.207 ± 0.036	111	0.448 ± 0.071
0.1 *μ*g/L BL	0.067 ± 0.062	0.155 ± 0.037	0.223 ± 0.036^*∗*^	120	0.458 ± 0.102
1 *μ*g/L BL	0.068 ± 0.008	0.156 ± 0.016	0.224 ± 0.020^*∗*^	121	0.440 ± 0.065
10 *μ*g/L BL	0.075 ± 0.009^*∗*^	0.159 ± 0.011^*∗*^	0.234 ± 0.023^*∗*^	126	0.470 ± 0.090^*∗*^
100 *μ*g/L BL	0.093 ± 0.008^*∗*^	0.181 ± 0.013^*∗*^	0.274 ± 0.038^*∗*^	148	0.526 ± 0.075^*∗*^

Data represent mean ± SD of three biological replicates and were tested for significance by Student's *t* test (*P* < 0.05). Asterisks indicate significant differences from the control (0 *μ*g/L BL).

**Table 2:** Effect of BL on biomass accumulation and root/shoot ratio of *in vitro* potato plants under NaCl stress.

Treatments	Biomass accumulation (g)			Relative biomass (%)	Root/shoot ratio
	Root fresh weight	Shoot fresh weight	Total plant fresh weight		
0 mM NaCl	0.095 ± 0.017^B^	0.217 ± 0.073^B^	0.312 ± 0.009^B^	100	0.438^C^
0 mM NaCl + BL	0.167 ± 0.103^A^	0.247 ± 0.089^A^	0.414 ± 0.008^A^	133	0.671^A^
50 mM NaCl	0.057 ± 0.014^C^	0.158 ± 0.045^D^	0.215 ± 0.004^D^	68.9	0.362^F^
50 mM NaCl + BL	0.070 ± 0.027^C^	0.173 ± 0.070^C^	0.243 ± 0.003^C^	77.9	0.407^E^
75 mM NaCl	0.034 ± 0.022^D^	0.078 ± 0.024^G^	0.112 ± 0.002^F^	35.9	0.440^D^
75 mM NaCl + BL	0.050 ± 0.021^C^	0.106 ± 0.022^E^	0.156 ± 0.008^E^	50.0	0.472^B^
100 mM NaCl	0.009 ± 0.110^E^	0.071 ± 0.045^H^	0.086 ± 0.003^G^	27.6	0.089^H^
100 mM NaCl + BL	0.011 ± 0.065^E^	0.093 ± 0.011^F^	0.104 ± 0.001^F^	33.3	0.118^G^

Data represent mean ± SD of three biological replicates and were tested for significance by Duncan's new multiple range test. Columns marked with different capital letters indicate significant differences (*P* < 0.01).

## References

[1] Rodziewicz P., Swarcewicz B., Chmielewska K., Wojakowska A., Stobiecki M. (2014). Influence of abiotic stresses on plant proteome and metabolome changes. *Acta Physiologiae Plantarum*.

[2] Sairam R. K., Tyagi A. (2004). Physiology and molecular biology of salinity stress tolerance in plants. *Current Science*.

[3] Abogadallah G. M. (2010). Insights into the significance of antioxidative defense under salt stress. *Plant Signaling and Behavior*.

[4] Khalid A., Aftab F. (2016). Effect of exogenous application of 24-epibrassinolide on growth, protein contents, and antioxidant enzyme activities of in vitro-grown *Solanum tuberosum* L. under salt stress. *In Vitro Cellular and Developmental Biology — Plant*.

[5] Gill S. S., Tuteja N. (2010). Reactive oxygen species and antioxidant machinery in abiotic stress tolerance in crop plants. *Plant Physiology and Biochemistry*.

[6] Miller G., Suzuki N., Ciftci-Yilmaz S., Mittler R. (2010). Reactive oxygen species homeostasis and signalling during drought and salinity stresses. *Plant, Cell and Environment*.

[7] Krasensky J., Jonak C. (2012). Drought, salt, and temperature stress-induced metabolic rearrangements and regulatory networks. *Journal of Experimental Botany*.

[8] Cuin T. A., Shabala S. (2007). Amino acids regulate salinity-induced potassium efflux in barley root epidermis. *Planta*.

[9] Cuin T. A., Shabala S. (2005). Exogenously supplied compatible solutes rapidly ameliorate NaCl-induced potassium efflux from barley roots. *Plant and Cell Physiology*.

[10] Eyidogan F., Öz M. T. (2007). Effect of salinity on antioxidant responses of chickpea seedlings. *Acta Physiologiae Plantarum*.

[11] Pan Y. Q., Guo H., Wang S. M. (2016). The photosynthesis, Na^+^/K^+^ homeostasis and osmotic adjustment of *Atriplex canescens* in response to salinity. *Frontiers in Plant Science*.

[12] Anschütz U., Becker D., Shabala S. (2014). Going beyond nutrition: regulation of potassium homoeostasis as a common denominator of plant adaptive responses to environment. *Journal of Plant Physiology*.

[13] Shabala S., Pottosin I. (2014). Regulation of potassium transport in plants under hostile conditions: implications for abiotic and biotic stress tolerance. *Physiologia Plantarum*.

[14] Vriet C., Russinova E., Reuzeaua C. (2012). Boosting crop yields with plant steroids. *The Plant Cell*.

[15] Divi U. K., Rahman T., Krishna P. (2016). Gene expression and functional analyses in brassinosteroid-mediated stress tolerance. *Plant Biotechnology Journal*.

[16] Divi U. K., Krishna P. (2009). Brassinosteroid: a biotechnological target for enhancing crop yield and stress tolerance. *New Biotechnology*.

[17] Deng X.-G., Zhu T., Zhang D.-W., Lin H.-H. (2015). The alternative respiratory pathway is involved in brassinosteroid-induced environmental stress tolerance in Nicotiana benthamiana. *Journal of Experimental Botany*.

[18] Bajguz A., Hayat S. (2009). Effects of brassinosteroids on the plant responses to environmental stresses. *Plant Physiology and Biochemistry*.

[19] Nakashita H., Yasuda M., Nitta T. (2003). Brassinosteroid functions in a broad range of disease resistance in tobacco and rice. *The Plant Journal*.

[20] Shu H., Ni W., Guo S. (2015). Root-applied brassinolide can alleviate the NaCl injuries on cotton. *Acta Physiologiae Plantarum*.

[21] Wang B., Zhang J., Xia X., Zhang W.-H. (2011). Ameliorative effect of brassinosteroid and ethylene on germination of cucumber seeds in the presence of sodium chloride. *Plant Growth Regulation*.

[22] Kagale S., Divi U. K., Krochko J. E., Keller W. A., Krishna P. (2007). Brassinosteroid confers tolerance in *Arabidopsis thaliana* and *Brassica napus* to a range of abiotic stresses. *Planta*.

[23] Zhang S., Hu J., Zhang Y., Xie X. J., Knapp A. (2007). Seed priming with brassinolide improves lucerne (*Medicago sativa* L.) seed germination and seedling growth in relation to physiological changes under salinity stress. *Australian Journal of Agricultural Research*.

[24] Ali B., Hayat S., Ahmad A. (2007). 28-Homobrassinolide ameliorates the saline stress in chickpea (*Cicer arietinum* L.). *Environmental and Experimental Botany*.

[25] Shahid M., Pervez M., Balal R. (2011). Brassinosteroid (24-Epibrassinolide) enhances growth and alleviates the deleterious effectsinduced by salt stress in pea (*Pisum sativum* L.). *Australian Journal of Crop Science*.

[26] Shahbaz M., Ashraf M. (2008). Does exogenous application of 24-epibrassinolide ameliorate salt induced growth inhibition in wheat (*Triticum aestivum* L.)?. *Plant Growth Regulation*.

[27] Sharma I., Ching E., Saini S., Bhardwaj R., Pati P. K. (2013). Exogenous application of brassinosteroid offers tolerance to salinity by altering stress responses in rice variety Pusa Basmati-1. *Plant Physiology and Biochemistry*.

[28] Krishna P. (2003). Brassinosteroid-mediated stress responses. *Journal of Plant Growth Regulation*.

[29] El-Mashad A. A. A., Mohamed H. I. (2012). Brassinolide alleviates salt stress and increases antioxidant activity of cowpea plants (*Vigna sinensis*). *Protoplasma*.

[30] Müssig C., Shin G.-H., Altmann T. (2003). Brassinosteroids promote root growth in *Arabidopsis*. *Plant Physiology*.

[31] González-García M.-P., Vilarrasa-Blasi J., Zhiponova M. (2011). Brassinosteroids control meristem size by promoting cell cycle progression in *Arabidopsis* roots. *Development*.

[32] Clouse S. D., Langford M., McMorris T. C. (1996). A brassinosteroid-insensitive mutant in *Arabidopsis thaliana* exhibits multiple defects in growth and development. *Plant Physiology*.

[33] Xiao L. T., Wang S. G. (2005). *Experimental Techniques of Plant Physiology*.

[34] Hodges D. M., DeLong J. M., Forney C. F., Prange R. K. (1999). Improving the thiobarbituric acid-reactive-substances assay for estimating lipid peroxidation in plant tissues containing anthocyanin and other interfering compounds. *Planta*.

[35] Bates L. S., Waldren R. P., Teare I. D. (1973). Rapid determination of free proline for water-stress studies. *Plant and Soil*.

[36] Pandey G. K., Cheong Y. H., Kim B.-G., Grant J. J., Li L., Luan S. (2007). CIPK9: a calcium sensor-interacting protein kinase required for low-potassium tolerance in *Arabidopsis*. *Cell Research*.

[37] Bao F., Shen J., Brady S. R., Muday G. K., Asami T., Yang Z. (2004). Brassinosteroids interact with auxin to promote lateral root development in *Arabidopsis*. *Plant Physiology*.

[38] Zenk M. H., Kasha K. J. (1974). Haploids in physiological and biochemical research. *Haploids in higher plants*.

[39] Khripach V., zhabinskii v., de Groot A. (2000). Twenty years of brassinosteroids: steroidal plant hormones warrant better crops for the XXI century. *Annals of Botany*.

[40] Xia X.-J., Huang L.-F., Zhou Y.-H. (2009). Brassinosteroids promote photosynthesis and growth by enhancing activation of Rubisco and expression of photosynthetic genes in *Cucumis sativus*. *Planta*.

[41] Mandava N. B. (1988). Plant growth-promoting brassinosteroids. *Annual Review of Plant Biology*.

[42] Habib S. H., Kausar H., Saud H. M. (2016). Plant growth-promoting rhizobacteria enhance salinity stress tolerance in okra through ROS-scavenging enzymes. *Biomed Research International*.

[43] Noreen Z., Ashraf M. (2009). Assessment of variation in antioxidative defense system in salt-treated pea (*Pisum sativum*) cultivars and its putative use as salinity tolerance markers. *Journal of Plant Physiology*.

[44] Wang C.-M., Zhang J.-L., Liu X.-S. (2009). Puccinellia tenuiflora maintains a low Na^+^ level under salinity by limiting unidirectional Na^+^ influx resulting in a high selectivity for K^+^ over Na^+^. *Plant, Cell and Environment*.

[45] Yamaguchi T., Blumwald E. (2005). Developing salt-tolerant crop plants: challenges and opportunities. *Trends in Plant Science*.

